# Cognitive processes and pathways between social isolation, loneliness, and paranoia: findings from a cross-lagged network analysis of population-based data

**DOI:** 10.1017/S003329172510130X

**Published:** 2025-07-28

**Authors:** Błażej Misiak

**Affiliations:** Department of Psychiatry, Wroclaw Medical University, Wroclaw, Poland

**Keywords:** cognitive bias, delusion, ideas of reference, persecutory thoughts, solitude

## Abstract

**Background:**

Social disconnection, covering loneliness and social isolation, might be associated with the development of paranoid thoughts. Differential effects of loneliness and social isolation on the occurrence of paranoia have not been tested so far. Moreover, the role of cognitive mechanisms in these associations remains unknown. This study aimed to investigate differential associations of loneliness and social isolation with paranoid thoughts in the general population, considering the role of cognitive mechanisms.

**Methods:**

Altogether, 3,275 individuals, enrolled from the general population, completed baseline and follow-up assessments spanning 6–7 months. Cognitive biases (rejection sensitivity, attributional biases, and safety behaviors), social cognitive problems, and subjective cognitive problems were measured. The cross-lagged panel network (CLPN) analysis was performed, controlling for the effects of sociodemographic characteristics, psychiatric treatment, substance use, depressive, and anxiety symptoms. Additionally, mediation was tested for the CLPN paths linking social disconnection with paranoid ideation, with one intermediary node representing cognitive processes.

**Results:**

Loneliness was the most important node in terms of predicting other network variables. It was bidirectionally associated with paranoid thoughts. Cognitive processes mediated these associations (partial mediation for ideas of reference and full mediation for ideas of persecution). In turn, social isolation predicted paranoid thoughts through the effects on loneliness. It was also predicted by paranoid thoughts through attributional biases.

**Conclusions:**

Social disconnection might be bidirectionally associated with paranoid thoughts. However, loneliness is more closely tied to paranoid thoughts compared to social isolation. Cognitive processes might mediate the association of social disconnection with paranoid thoughts.

## Introduction

The term ‘paranoia’ refers to thoughts of excessive mistrust and suspicion toward other people. These thought impairments lie on the continuum that extends from mild manifestations, frequently observed in the general population sample, to paranoid delusions associated with a high level of conviction in thoughts, distress, and reduced social functioning (Bebbington et al., 2013; Freeman et al., 2005). It has been shown that the latent structure of paranoia is rather dimensional than categorical across clinical and nonclinical samples (Edens, Marcus, & Morey, 2009). Therefore, dimensional approaches are needed to better understand the mechanisms underlying the occurrence and progression of paranoid ideation.

Contextual processes are not only the content of paranoid thoughts but also play an important role in their development (Jester et al., 2023). Over recent years, some studies have focused on the role of social disconnection as both the cause and consequence of paranoia (Fulford & Holt, 2023). Social disconnection is usually understood in light of two constructs, that is, social isolation and loneliness. The first one serves as an objective phenomenon referring to a low number of social contacts. The second one is subjective and occurs when individual social bonds are self-perceived as insufficient. A recent meta-analysis of cross-sectional studies demonstrated that psychotic experiences, especially paranoia, are associated with loneliness (Chau, Zhu, & So, 2019). However, little is known about longitudinal associations that have been studied for psychotic experiences in general, but not paranoia specifically (Endo et al., 2022; Qiao et al., 2024). Experimental recollection of loneliness has been found to increase the level of paranoia, supporting causal effects (Gollwitzer, Wilczynska, & Jaya 2018; Lamster, Nittel, Rief, Mehl, Lincoln 2017). Some insights have also been provided by the experience sampling method studies, showing that individuals with psychosis tend to spend time alone and experience higher levels of paranoia while being alone (Fett et al., 2022). It has also been observed that momentary isolation from others and loneliness might predict the emergence of psychotic experiences in daily life (Bell et al., 2023; Chau, So, & Barkus, 2023; Misiak et al., 2024).

Evolutionary frameworks posit that social interactions are necessary to increase the chances of reproduction and survival by providing a greater defensive capacity (Cacioppo, Cacioppo, & Boomsma, 2014; Cacioppo & Hawkley, 2009). Taking into consideration these benefits, humans show an inherent ability to form social connections. In this context, the experience of loneliness is aversive but might be adaptive while signaling the need to activate cognitive processes and behaviors that aim to reconnect with others (Cacioppo & Hawkley, 2009). However, some individuals who experience loneliness do not activate processes and behaviors that aim to restore social connections. They tend to neglect opportunities to reconnect and approach a defensive cognitive style by focusing on social cues that inform them about potential threats from others. This strategy is supposed to be defensive as it aims to protect against threats associated with being alone. These individuals are likely to show a number of cognitive biases, including hypervigilance to social threats, attributional biases, expectations of rejection from others, negative perceptions of self and others, the focus on prevention-oriented goals, and low self-efficacy (Spithoven, Bijttebier, & Goossens, 2017). Eventually, these cognitive biases further contribute to social disconnection through a self-defeating loop.

Cognitive biases and impairments of social cognition have been widely documented among individuals with psychosis and those at clinical high risk of psychosis (Gaweda et al., 2024; van Donkersgoed et al., 2015; Weinreb, Li, & Kurtz, 2022). Also, there is evidence that cognitive biases are associated with psychotic experiences in nonclinical samples (Livet, Navarri, Potvin, & Conrod, 2020). From a clinical perspective, impairments across cognitive processes are recognized as potential targets for therapeutic interventions (Nijman, Veling, van der Stouwe, & Pijnenborg, 2020; Sauve et al., 2020). Although the evolutionary theory of loneliness has largely improved our understanding of cognitive processes involved in the development and progression of loneliness, it remains unknown whether it might be relevant for understanding the occurrence of paranoid ideation in the general population. It is also unknown whether loneliness and social isolation differ in terms of their associations with psychosis. Complex processes related to psychopathology are increasingly being addressed using network models that recognize their dynamic associations over time and provide insights into the relative importance of concurrent phenomena by estimating centrality metrics (Borsboom et al., 2021). Recent advancements in network modeling have provided a novel approach, known as the cross-lagged panel network (CLPN) analysis (Wysocki, van Bork, Cramer, & Rhemtulla, 2022). This approach provides opportunities to model longitudinal associations while controlling for autoregressive processes without the use of a predefined causal conceptualization. Taking into consideration existing research gaps and recent advancements in the development of network models, the present study had two aims. First, this study aimed to investigate whether cognitive processes mediate the longitudinal association of social disconnection with paranoid ideations in the general population. Second, it aimed to disentangle whether social isolation and loneliness differ in terms of their associations with paranoia in the general population sample.

## Methods

### Participants

Recruitment procedures were implemented by means of the quota sampling to ensure sample representativeness with respect to sociodemographic characteristics (age, gender, the level of education, employment status, and place of residence). Baseline assessments were performed using the internet-based survey between July and August 2024. Some results from the baseline assessment were published previously (Misiak, 2024). The follow-up assessments were carried out in February 2025. Recruitment was performed by a research company using its own online access panel of registered participants. Members of this panel are enrolled by means of regular campaigns. For underrepresented groups (e.g. old-age individuals and ethnic minorities), additional recruitment campaigns are regularly initiated. All panel members aged at least 18 years were considered eligible. The participants were informed about the confidentiality of all assessments. They provided informed consent to participate in this study. The study received approval from the Bioethics Committee at Wroclaw Medical University, Wroclaw, Poland (Approval number: 553/2024).

### Measures

*Loneliness:* To measure loneliness, the 11-item version of De Jong Gierveld Loneliness Scale (DJGLS) was administered (de Jong-Gierveld & Kamphuls, 1985; Grygiel et al., 2013). The items include five possible responses (“yes!,” “yes,” “more or less,” “no,” and “no!”). There are two subscales of the DJGLS. The first one refers to emotional aspects of loneliness (six items). In turn, the second one measures social aspects of loneliness (five items). The total loneliness score is calculated by summing positive and neutral responses (“yes!,” “yes,” and “more or less”) across items measuring emotional loneliness together with negative and neutral responses (“no!,” “no,” and “more or less”) to those referring to social loneliness. The total loneliness score ranges between 0 and 11. Higher scores reflect a greater level of loneliness. In this study, the Cronbach’s alphas for emotional and social loneliness subscales were 0.874 and 0.810, respectively.

*Social isolation:* To measure the level of social isolation, the six-item version of the Lubben Social Network Scale (LSNS-6) was used (Lubben et al., 2006). Specific items record the number of family members and friends who are seen or heard at least once a month, with whom the respondent can talk about private matters, and who can be called on for help. Each item is based on a 6-point scale. The total score ranges between 0 and 20. Higher scores reflect a greater network of social contacts. To ease the interpretation of findings and show the level of social isolation, the total LSNS-6 was reversed. The Cronbach’s alpha of the LSNS-6 was 0.876 in the present study.

*Cognitive processes:* In the present study, the 18-item version (Gaweda et al., 2018) of the Davos Assessment of Cognitive Biases (DACOBS-18) (van der Gaag et al., 2013) was administered. Each item is based on a 7-point scale (1 – “strongly disagree” and 7 – “strongly agree”). The items measure two cognitive biases (attributional biases and safety behaviors), social cognition problems, and subjective cognitive problems. In study, the Cronbach’s alphas were as follows: 0.801 for attributional biases, 0.745 for safety behaviors, 0.733 for social cognition problems, and 0.809 for subjective cognitive problems.

In turn, to assess rejection sensitivity, the Adult Rejection Sensitivity Questionnaire was administered (Berenson et al., 2009). It is based on nine vignettes describing various social contexts. The respondent is asked to imagine the involvement in each situation and the rejection concern (1 – “very unconcerned” and 6 – “very concerned”) and rejection expectancy (1 – “very unlikely” and 6 – “very likely”). For each vignette, the rejection sensitivity score is estimated by multiplying the rejection concern score by the rejection expectancy score. Next, the mean across all vignettes is estimated, representing the total rejection sensitivity score. In this study, the Cronbach’s alphas were 0.868 for the rejection concern subscale and 0.826 for the rejection expectancy subscale.

*Paranoid ideation:* To assess paranoid ideation, the Revised Green et al. Paranoid Thoughts Scale (R-GPTS) was used (Freeman et al., 2021). The R-GPTS items represent two subscales recording ideas of reference and ideas of persecution in the preceding month. Each item includes a 5-point scale (0 – “not at all” and 4 – “totally”). The items cover the experiences that did not appear as the effects of substance use. The optimal threshold scores for discriminating clinically relevant symptoms are ≥16 for ideas of reference and ≥11 for ideas of persecution (Freeman et al., 2021). In this study, the Cronbach’s alphas were 0.930 for ideas of reference and 0.964 for ideas of persecution.

*Depressive symptoms:* To measure depressive symptoms, the Patient Health Questionnaire-9 (PHQ-9) was used (Kroenke, Spitzer, & Williams, 2001). The PHQ-9 measures depressive symptoms in the preceding 2 weeks. The items are based on a 4-point scale (0 – “not at all” and 3 – “nearly every day”). The total score is 0–27 (higher scores refer to a greater level of depressive symptoms). The optimal threshold score for discriminating clinically relevant depressive symptoms has been estimated at ≥10 (Negeri et al., 2021). In this study, the Cronbach’s alpha was 0.897.

*Anxiety symptoms:* Anxiety symptoms were assessed using the Generalized Anxiety Disorder-7 (GAD-7) (Spitzer, Kroenke, Williams, & Lowe, 2006). The GAD-7 is based on seven items (rated on 4-point scale: 0 – “not at all” and 3 – “nearly every day”) that refer to the presence of generalized anxiety symptoms in the preceding 2 weeks. Items are rated on a 4-point scale (0 – “not at all” and 3 – “nearly every day”). The total score is 0–21 (higher scores indicate a greater level of anxiety symptoms). The optimal threshold score for discriminating clinically relevant generalized anxiety has been estimated at ≥10 (Spitzer et al., 2006). In this study, the Cronbach’s alpha was 0.944.

### Data analysis

Individuals who completed assessments at both time points (further referred to as completers) and those who did not complete the follow-up assessment (further referred to as non-completers) were compared with respect to sociodemographic characteristics, symptom profiles, cognitive biases, social cognition, and subjective cognitive problems using the *χ* ^2^-test (categorical variables) and *t*-tests (continuous variables). Differences between both groups were considered significant if the *p*-value < 0.05. These analyses were performed in the JASP software.

Next, a two-step approach to analyze the data was followed. In the first step, the data were analyzed using CLPN models. Next, specific mediation models were analyzed. This mixed-model design was used as a formal analysis of mediation cannot be performed using CLPN. However, CLPN analysis does not impose a strict causal structure (Borsboom et al., 2021), and thus it might inform more focused models generating specific hypotheses about mediation. The CLPN and mediation models were analyzed among the study completers. No missing data were present in this group.

The following variables were analyzed with respect to cross-lagged and autoregressive effects using CLPN models: (1) loneliness; (2) social isolation; (3) attributional biases; (4) safety behaviors; (5) rejection sensitivity; (6) social cognition problems; (7) subjective cognitive problems; (8) ideas of reference; and (9) ideas of persecution. Age, gender, the level of education, employment status, monthly income, a lifetime history of psychiatric treatment, substance use in the preceding month (except for nicotine and alcohol), and baseline depressive and anxiety symptoms were used as covariates. The least absolute shrinkage and selection operator regressions were used to estimate autoregressive and cross-lagged effects to avoid indicating weak associations (Tibshirani, 1996). The CLPN was modeled in the R Studio. Regressions were analyzed in the *glmnet* package (Friedman, Hastie, & Tibshirani, 2010). In turn, the *qgraph* package was used to visualize the results (Epskamp et al., 2012).

To assess the importance of specific nodes in the network, strength centrality was estimated (Jones, Mair, & McNally, 2018). It shows the sum of edge weights between a specific node and other nodes in the network. This centrality metric is most commonly used in network models of psychotic experiences in community samples (Misiak, Pytel, & Stanczykiewicz, 2025). In the case of CLPN, two categories of centrality are analyzed, that is, the output and input centrality. Out-strength shows the sum of edge weights from a specific node to all other nodes in the network. In turn, in-strength refers to the sum of edge weights to a specific node from all other nodes in the network.

Finally, nonparametric and case-drop bootstrapping was performed to analyze the network accuracy and stability in the *bootnet* package (Epskamp, Borsboom, & Fried, 2018). These analyses provide the correlation stability coefficient (CS-C). It shows the maximal proportion of data that can be dropped to retain the correlation coefficient of >0.70 between data from the original sample and the data retained. The network is considered stable if the CS-C is higher than 0.25 (Epskamp & Fried, 2018). Bootstrapping was also used to estimate the significance of differences between centrality metrics and edge weights.

Direct and indirect paths (with one intermediary node) between the nodes representing social disconnection (either loneliness or social isolation) and paranoid ideation (ideas of reference and ideas of persecution) were analyzed. Additional analyses of mediation were performed using the PROCESS macro (Model 4) (Hayes, 2018). Specific models were tested for the paths where one intermediary node representing cognitive processes connected either loneliness or social isolation with ideas of reference or ideas of persecution in the CLPN model. Results of these analyses were interpreted as significant if the 95% confidence interval did not include the zero value.

## Results

### Descriptive characteristics of the sample

The flow diagram of participants is shown in Supplementary Figure S1. Altogether, 5,099 individuals completed the baseline assessment (aged 44.9 ± 15.4, 47.7% men). In turn, a total of 3,275 participants completed the follow-up assessment, resulting in the study retention rate of 64.2%. Follow-up completers and non-completers did not differ significantly across sociodemographic characteristics and all other measures used in the present study (Table 1).

**Table 1.** Descriptive characteristics of the sample

	Total sample (*n* = 5,099)	Completers (*n* = 3,275)	Non-completers (*n* = 1,824)	*p*
Age, years	44.9 ± 15.4	45.2 ± 15.7	44.8 ± 15.2	0.189
Gender, men	2,431 (47.7)	1,568 (47.9)	863 (47.3)	0.718
Education Primary Vocational Secondary Higher	94 (1.8) 411 (8.1) 2,098 (41.1) 2,496 (49.0)	53 (1.6) 273 (8.3) 1,345 (41.1) 1,604 (49.0)	41 (2.2) 138 (7.6) 753 (41.3) 892 (48.9)	0.858
Employment Unemployed Retired/disability pension Student Part-time work Full-time work	435 (8.5) 1,002 (19.6) 234 (4.6) 482 (9.5) 2,946 (57.8)	265 (8.1) 632 (19.3) 156 (4.8) 317 (9.7) 1,905 (58.1)	170 (9.3) 370 (20.3) 78 (4.3) 165 (9.0) 1041 (57.1)	0.406
Place of residence Rural Urban, <50,000 inhabitants Urban, 50,000–150,000 inhabitants Urban, 150,000–500,000 inhabitants Urban, >500,000 inhabitants	1,650 (32.4) 1,166 (22.9) 816 (16.0) 714 (14.0) 753 (14.7)	1,065 (32.5) 750 (22.9) 525 (16.0) 457 (14.0) 478 (14.6)	585 (32.1) 416 (22.8) 291 (16.0) 257 (14.0) 275 (15.1)	0.991
Monthly income <750 USD 750–1500 USD 1,500–2,500 USD 2,500–3,750 USD >3,750 USD Refused to answer	1,082 (21.1) 2,485 (48.7) 722 (14.2) 133 (2.6) 44 (0.9) 633 (12.4)	695 (21.2) 1,621 (49.6) 468 (14.3) 80 (2.4) 31 (0.9) 380 (11.6)	387 (21.2) 864 (47.4) 254 (13.9) 53 (2.9) 13 (0.7) 253 (13.9)	0.170
Psychiatric treatment Lifetime Preceding month	1,108 (21.7) 539 (10.6)	705 (21.5) 327 (10.0)	403 (22.1) 512 (28.1)	0.638 0.068
Substance use, preceding month^a^	353 (6.9)	213 (6.5)	140 (7.7)	0.353
Depressive symptoms, PHQ–9 Total score ≥10^b^	7.4 ± 6.0 1,533 (30.1)	7.3 ± 5.8 954 (29.1)	7.6 ± 6.1 574 (31.5)	0.078 0.080
Anxiety symptoms, GAD–7 Total score ≥10^b^	6.1 ± 5.5 1,205 (23.6)	5.9 ± 5.3 747 (22.8)	6.2 ± 5.6 458 (25.1)	0.068 0.064
Ideas of reference, R-GPTS Total score ≥16^b^	9.1 ± 8.1 1,258 (24.7)	9.3 ± 8.9 818 (25.0)	9.5 ± 7.9 440 (24.1)	0.411 0.498
Ideas of persecution, R-GPTS Total score ≥11^b^	7.7 ± 10.0 1,527 (29.9)	8.0 ± 10.5 967 (29.5)	8.1 ± 9.7 560 (30.7)	0.443 0.380
Rejection sensitivity, ARSQ	11.5 5.3	11.5 ± 5.4	11.6 ± 5.2	0.383
Subjective cognitive problems, DACOBS	16.5 ± 5.9	16.3 ± 5.6	16.5 ± 6.1	0.301
Social cognition problems, DACOBS	14.2 ± 4.4	14.0 ± 4.2	14.1 ± 4.5	0.211
Attributional biases, DACOBS	13.9 ± 5.0	13.8 ± 5.0	14.0 ± 5.0	0.091
Safety behaviors, DACOBS	14.4 ± 5.5	14.3 ± 5.4	14.5 ± 5.6	0.126
Social isolation, LSNS–6^c^	15.9 ± 6.0	16.0 ± 6.5	15.8 ± 5.8	0.228
Loneliness, DJGLS	5.2 ± 3.9	5.1 ± 3.7	5.3 ± 4.0	0.104

*Note:* Data are reported as mean ± SD or *n* (%).

Abbreviations: ARSQ, Adult Rejection Sensitivity Questionnaire; DACOBS, Davos Assessment of Cognitive Biases Scale; DJGLS, De Jong Gierveld Loneliness Scale; GAD-7, Generalized Anxiety Disorder-7; LSNS-6, Lubben Social Network Scale-6; PHQ-9, Patients Health Questionnaire-9; R-GPTS, the Revised Green et al. Paranoid Thoughts Scale.

a Except for nicotine and alcohol.

b Optimal thresholds for discriminating clinically relevant symptoms (see the main text).

c Reversed coding was used to show the level of social isolation.

### Network analysis

**Figure 1.**
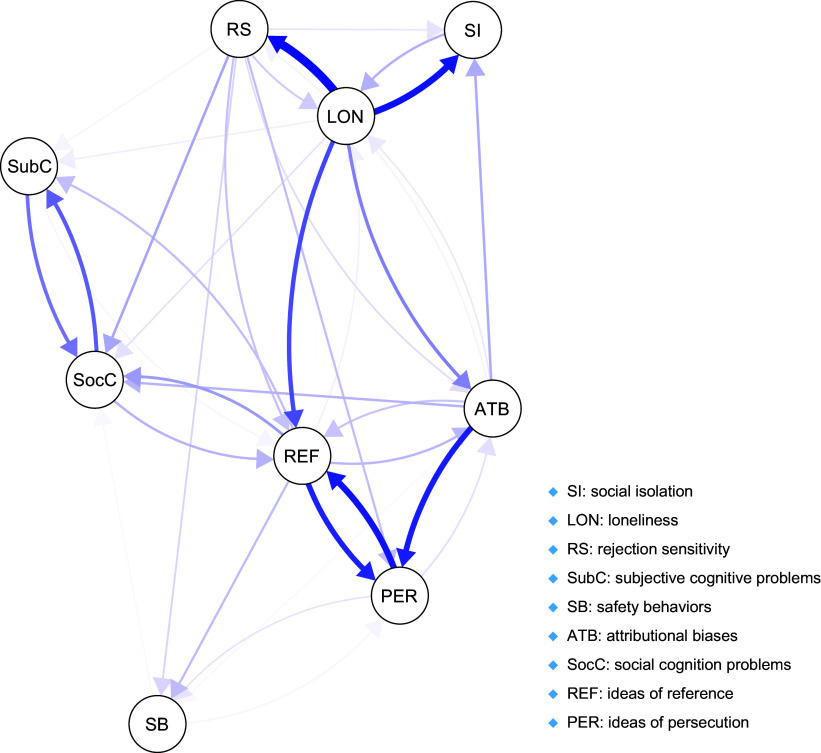
The network estimated in the present study. Arrows show cross-lagged associations. Thicker and more saturated arrows refer to stronger cross-lagged effects. To ease visual interpretation, autoregressive effects were removed.

**Figure 2.**
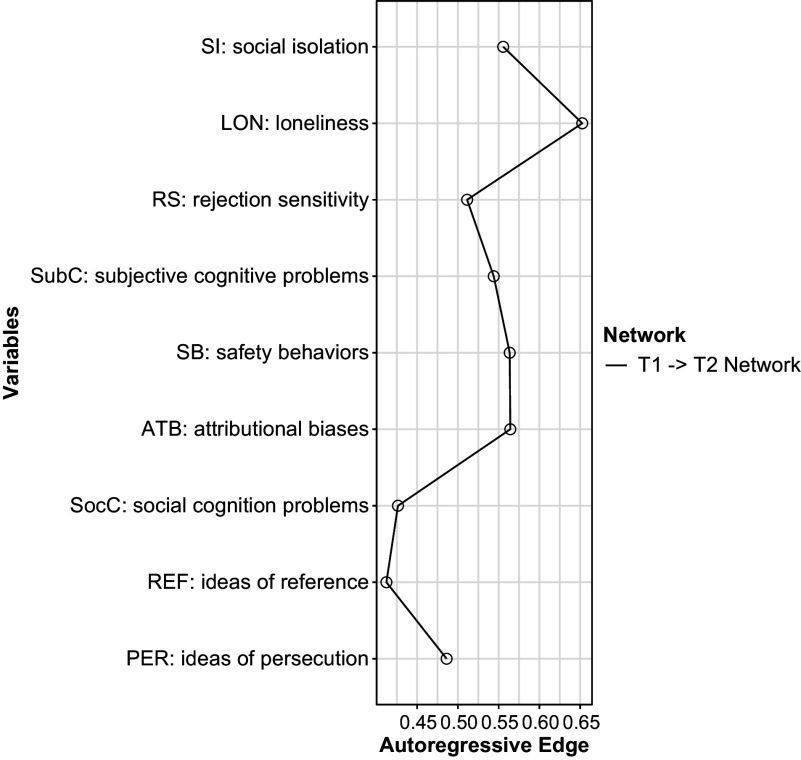
Autoregressive effects across variables measured in the present study.

*Overview of the network:* The network estimated in the present study is visualized in Figure 1. There were 38 nonzero cross-lagged edges (46.9%). Negative edges (i.e. those showing negative correlations) were not found. Autoregressive effects are shown in Figure 2. The highest autoregressive effect was observed for loneliness, while the lowest one was found for ideas of reference. The strongest cross-lagged effect led from loneliness to rejection sensitivity, while the weakest one was from ideas of reference to rejection sensitivity (Supplementary Figure S2). The connection from loneliness to rejection sensitivity was significantly stronger compared to almost all cross-lagged associations in the network (except for the effect of loneliness on social isolation, as well as cross-lagged effects between ideas of persecution and ideas of reference).

*Paths from social disconnection to paranoia:* Loneliness directly predicted ideas of reference, but not ideas of persecution. Other paths from loneliness to ideas of persecution, with one intermediary node, led through ideas of reference, rejection sensitivity, and attributional biases. Also, other paths (with one intermediary node representing cognitive processes) from loneliness to ideas of reference led through rejection sensitivity, subjective and social cognition problems, and attributional biases.

Social isolation directly predicted loneliness; however, it did not directly predict ideas of reference and ideas of persecution. The path from social isolation to ideas of reference led through the effects of loneliness. There were no paths leading from social isolation to ideas of persecution through one intermediary node.

*Paths from paranoia to social disconnection:* Ideas of reference, but not ideas of persecution, directly predicted loneliness. This effect was not significantly stronger compared to the reversed one (i.e. from loneliness to ideas of reference; Supplementary Figure S2). The paths from ideas of persecution to loneliness with one intermediary node led through ideas of reference and attributional biases. In turn, the paths from ideas of reference to loneliness with one intermediary node led through attributional biases, but not through other cognitive processes.

Social isolation was directly predicted by loneliness but not by paranoid thoughts (either ideas of reference or ideas of persecution). The path from ideas of reference to social isolation with one intermediary node led through loneliness and attributional biases. In turn, the path from ideas of persecution to social ideation with one intermediary node led through attributional biases.

**Figure 3.**
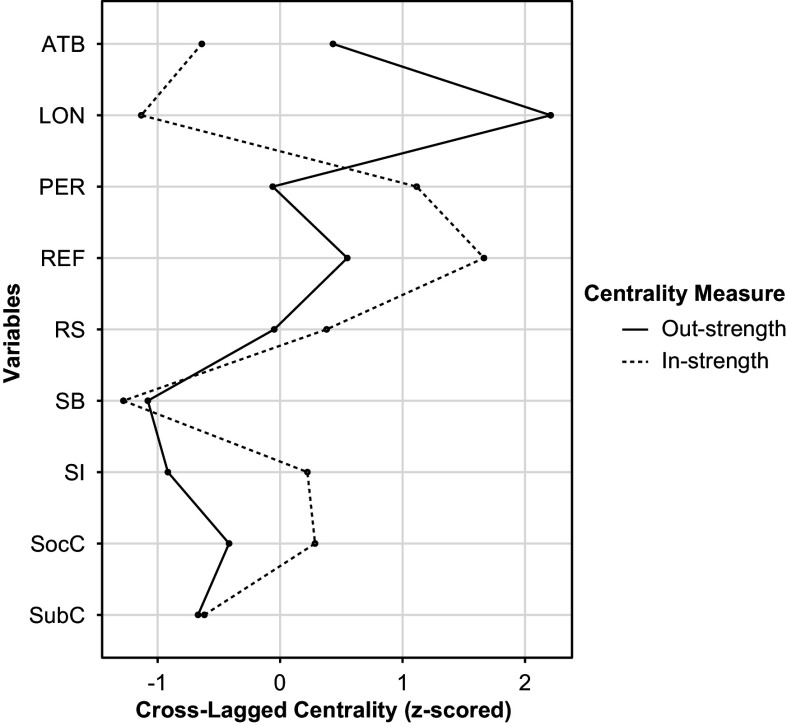
The strength centrality metrics.

*Strength centrality:* The highest out-strength centrality was found for loneliness (Figure 3). It was significantly higher compared to all other nodes in the network (Supplementary Figure S3). The highest in-strength centrality was identified for ideas of reference. It was significantly higher than the in-strength centrality of loneliness, safety behaviors, attributional biases, and subjective cognitive problems (Supplementary Figure S4).

*Network stability and accuracy:* The CS-C values were as follows: 0.672 for edges, 0.595 for out-strength centrality, and 0.439 for in-strength centrality. Therefore, the network might be interpreted as stable while retaining various proportions of data. Results of stability and accuracy analyses are visualized in Supplementary Figures S5 and S6.

### Mediation analyses

There were significant effects of baseline loneliness on follow-up ideas of reference (Supplementary Table S2). Vice versa, baseline levels of ideas of reference directly predicted follow-up levels of loneliness. Indirect effects of cognitive processes (i.e. attributional biases, subjective cognitive problems, social cognition problems, and rejection sensitivity) in these associations were also significant, indicating the presence of partial mediation. Direct associations of loneliness with ideas of persecution with loneliness were not significant. However, significant indirect effects of cognitive processes in the association of loneliness with ideas of persecution were found, indicating the presence of full mediation.

## Discussion

Findings from the present study imply that loneliness might be bidirectionally associated with the occurrence of paranoid thoughts, especially ideas of reference. However, loneliness might be more closely related to paranoid thoughts. It also had the greatest importance in terms of predicting other variables in the network. The observations extend the results of a recent meta-analysis showing that psychotic experiences are cross-sectionally associated with loneliness across the psychosis continuum, with stronger and more robust correlations observed for paranoia (Chau et al., 2019). Previous studies have also shown that loneliness and social isolation are modestly correlated, suggesting different underlying mechanisms (Cacioppo & Hawkley, 2009; Cacioppo, Hawkley, & Bernston, 2003). It is also needed to note that while loneliness is always a distressing experience, social isolation might be congruent with individual social needs. A recent study, based on the experience sampling method, revealed that momentary social isolation does not exert negative effects on emotional responses when it is autonomous (choiceful) and does not accumulate over days (Weinstein, Vuorre, Adams, & Nguyen, 2023). Other studies have shown that the preference for social isolation might be a strategy to regulate high-arousal and negative emotions (Nguyen, Konu, & Forbes, 2025; Nguyen, Ryan, & Deci, 2018). Finally, it has been suggested that social isolation might provide opportunities for the development of self-knowledge and self-connection that are difficult to achieve while spending time with others (Long & Averill, 2003).

It is also important to note that ideas of reference were more closely related to loneliness than ideas of persecution. Indeed, the latter dimension of paranoid thoughts was not found to be directly associated with loneliness. Cognitive processes, especially attributional biases, appeared to be more closely tied to the association between loneliness and ideas of persecution (full mediation) than the one between loneliness and ideas of reference (partial mediation). In addition to attributional biases, other cognitive biases may play an important role in explaining the associations between social disconnection and paranoid ideation. The present study included rejection sensitivity, a cognitive-affective bias that reflects heightened expectations of social rejection and exaggerated concern about its occurrence (Berenson et al., 2009). Rejection sensitivity has been previously implicated in the emergence and maintenance of paranoia, particularly among individuals with psychosis and those at risk (Kesting & Lincoln, 2013; Lincoln, Johnson, Winters, & Laquidara, 2021). In the present study, rejection sensitivity was found to mediate the association of loneliness with ideas of reference. This finding aligns with theoretical models suggesting that loneliness may activate maladaptive cognitive-affective processes, such as heightened rejection sensitivity, which in turn amplify mistrust and threat-related interpretations of social interactions (Spithoven et al., 2017).

From a clinical perspective, these results underscore the potential value of addressing cognitive biases, including rejection sensitivity and attributional biases, in psychological interventions aimed at reducing paranoid ideation and loneliness. Previous meta-analyses have shown that psychological interventions might be effective in reducing the level of loneliness, albeit with low-to-medium effect size estimates (Hickin et al., 2021; Masi, Chen, Hawkley, & Cacioppo, 2011). The meta-analysis by Masi et al., (2011) concluded that social cognition interventions have the greatest effectiveness in reducing loneliness. In turn, in the meta-analysis by Hickin et al. (2021), cognitive-behavioral therapy (CBT), targeting perceptual and cognitive biases that result in hypervigilance to negative social information, was the most common intervention for loneliness. Potential effectiveness of CBT might be related to the fact that this intervention encourages individuals to seek out disconfirming evidence to challenge their perceptions of loneliness and enhance self-efficacy, with the goal of changing behaviors, building social connections, and ultimately reducing loneliness (Hickin et al., 2021; Kall et al., 2020).

Furthermore, it is important to note that CBT might be effective for reducing general and positive symptoms of psychosis with small-to-medium effect size estimates (Berendsen et al., 2024). However, evidence with respect to the efficacy of CBT in reducing the level of loneliness among individuals with psychosis and its subclinical presentations is scarce. To date, only one study investigated whether the state-of-the-art CBT for psychosis might be beneficial in terms of reducing the levels of loneliness (Winkler et al., 2024). However, caution is warranted when considering the extent to which the findings obtained from individuals with psychotic disorders (Berendsen et al., 2024; Winkler et al., 2024) correspond to those from the present study, conducted in a nonclinical cohort. The authors found significant symptom reductions during this treatment; however, changes in the levels of loneliness were not significant. Nevertheless, it is important to note that the CBT protocol assessed in this study did not include a specific module targeting loneliness.

Limitations of the study by Winkler et al. (2024) might also be related to using only one item that directly recorded the feelings of loneliness, pointing to the discussion on how loneliness should be measured. Although single-item measures are commonly used to record loneliness, they tend to provide lower prevalence rates compared to those obtained by studies based on indirect indicators, for example, DJGLS (Stegen et al., 2024). This discrepancy might be related to the observation that individuals with loneliness tend to underreport this experience (van den Broek, Lam, & Potente, 2024). This phenomenon might be related to the stigma of loneliness (Fan et al., 2025; Ko, Wei, Rivas, & Tucker, 2022; Shiovitz-Ezra & Ayalon, 2012).

The observation that cognitive mechanisms are not critical in the association between loneliness and paranoia indicates that other psychological processes might also be relevant. For instance, there is evidence that loneliness might be perceived as a stressful experience associated with a greater likelihood to experience negative emotions and physiological responses (Cacioppo et al., 2003; Luo & Shao, 2023; Steptoe, Owen, Kunz-Ebrecht, & Brydon, 2004). Previous studies have also shown that individuals with psychosis and those at risk of psychosis show increased stress sensitivity, manifesting in elevated emotional responses to minor daily stressors (Myin-Germeys & van Os, 2007). Impaired emotion regulation has also been observed among individuals experiencing loneliness (Patrichi, Rimbu, Miu, & Szentagotai-Tatar, 2025). In this regard, the models considering the relevance of both cognitive and emotional processes might provide further insights into the paths between social isolation, loneliness, and paranoia.

The present study is characterized by some limitations. The sample should be interpreted as nonclinical; therefore, translation of findings into clinical contexts needs to be approached with caution. At this point, it is also needed to note that the level of paranoid ideation was not validated using in-person clinical assessments. Moreover, the study did not record the full spectrum of cognitive biases related to the psychosis spectrum disorders, for example, jumping to conclusions and belief inflexibility. It is also needed to note that impairments across cognitive processes were not assessed using objective measures. This might be of importance for two domains of DACOBS, that is, subjective cognitive problems and social cognition problems. Another limitation is that a potential selection bias related to sampling among internet users cannot be excluded. Also, the cohort retention rate of 64.2% was relatively low. Furthermore, it should be noted that the data collection was based on two time points and, thus, insights into the temporal ordering of mediating effects of cognitive processes might be limited. Indeed, in all mediation models, either predictors and mediators or mediators and outcome variables were measured at the same time point. The limitations related to network modeling, where the results might depend on the inclusion of specific nodes, should also be considered. Finally, the study was observational and, thus, it informs about a temporal ordering of variables rather than causal effects.

In conclusion, the findings indicate that social disconnection, especially loneliness, is bidirectionally associated with paranoid thoughts in the general population. Altered information processing might link loneliness and social isolation with paranoia in some individuals. Future studies need to further investigate the mechanisms explaining the association of loneliness and social isolation with paranoia, beyond those related to cognitive processes. It is also needed to replicate the findings in clinical and general population cohorts with longer observation periods, as well as clinical trials of interventions targeting cognitive mechanisms associated with psychosis.

## Supporting information

Misiak supplementary material Misiak supplementary material

## Supplementary material

The supplementary material for this article can be found at http://doi.org/10.1017/S003329172510130X.
