# Supplementary material for: Cognitive processes and pathways between social isolation, loneliness, and paranoia: findings from a cross-lagged network analysis of population-based data
Source: Psychol Med. 2025 Jul 28;55:e215. doi: 10.1017/S003329172510130X (PMC12315646; doi:10.1017/S003329172510130X)
Supplement: Misiak supplementary material [file S003329172510130Xsup001.docx]

**Supplementary Appendix**

**Table S1.** Weights of non-zero, cross-lagged edges.

| nodeOut | nodeIn | value |
| --- | --- | --- |
| LON: loneliness | RS: rejection sensitivity | 0.164 |
| LON: loneliness | SI: social isolation | 0.119 |
| PER: ideas of persecution | REF: ideas of reference | 0.114 |
| ATB: attributional biases | PER: ideas of persecution | 0.101 |
| REF: ideas of reference | PER: ideas of persecution | 0.095 |
| LON: loneliness | REF: ideas of reference | 0.073 |
| SocC: social cognition problems | SubC: subjective cognitive problems | 0.064 |
| SubC: subjective cognitive problems | SocC: social cognition problems | 0.054 |
| LON: loneliness | ATB: attributional biases | 0.048 |
| REF: ideas of reference | SocC: social cognition problems | 0.037 |
| RS: rejection sensitivity | SocC: social cognition problems | 0.032 |
| ATB: attributional biases | SI: social isolation | 0.030 |
| SI: social isolation | LON: loneliness | 0.028 |
| ATB: attributional biases | SocC: social cognition problems | 0.028 |
| SocC: social cognition problems | REF: ideas of reference | 0.028 |
| REF: ideas of reference | ATB: attributional biases | 0.026 |
| REF: ideas of reference | SB: safety behaviors | 0.024 |
| REF: ideas of reference | SubC: subjective cognitive problems | 0.023 |
| RS: rejection sensitivity | PER: ideas of persecution | 0.023 |
| RS: rejection sensitivity | REF: ideas of reference | 0.022 |
| ATB: attributional biases | REF: ideas of reference | 0.021 |
| RS: rejection sensitivity | LON: loneliness | 0.019 |
| RS: rejection sensitivity | SB: safety behaviors | 0.015 |
| RS: rejection sensitivity | ATB: attributional biases | 0.012 |
| PER: ideas of persecution | ATB: attributional biases | 0.012 |
| LON: loneliness | SocC: social cognition problems | 0.011 |
| PER: ideas of persecution | SB: safety behaviors | 0.010 |
| RS: rejection sensitivity | SI: social isolation | 0.010 |
| ATB: attributional biases | LON: loneliness | 0.010 |
| LON: loneliness | SubC: subjective cognitive problems | 0.008 |
| REF: ideas of reference | LON: loneliness | 0.006 |
| ATB: attributional biases | RS: rejection sensitivity | 0.006 |
| SubC: subjective cognitive problems | REF: ideas of reference | 0.005 |
| RS: rejection sensitivity | SubC: subjective cognitive problems | 0.005 |
| SB: safety behaviors | PER: ideas of persecution | 0.004 |
| SB: safety behaviors | SocC: social cognition problems | 0.003 |
| ATB: attributional biases | SB: safety behaviors | 0.003 |
| REF: ideas of reference | RS: rejection sensitivity | 0.001 |

**Table S2.** Mediating effects of cognitive processes in the association of loneliness with ideas of reference and ideas of persecution.

| Model | Effect | b | SE | 95%CI |
| --- | --- | --- | --- | --- |
| LON (T0) → COG (T1) → REF (T1) | LON → ATB | 0.536 | 0.021 | 0.494 – 0.577 |
|  | LON → SocC | 0.386 | 0.019 | 0.349 – 0.424 |
|  | LON → SubC | 0.470 | 0.027 | 0.418 – 0.523 |
|  | LON → RS | 0.686 | 0.024 | 0.639 – 0.734 |
|  | LON → REF | 0.211 | 0.040 | 0.133 – 0.289 |
|  | ATB → REF | 0.103 | 0.027 | 0.050 – 0.156 |
|  | SocC → REF | 0.258 | 0.041 | 0.177 – 0.339 |
|  | SubC → REF | 0.208 | 0.028 | 0.153 – 0.263 |
|  | RS → REF | 0.103 | 0.027 | 0.050 – 0.156 |
|  | LON → ATB → REF | 0.180 | 0.022 | 0.138 – 0.224 |
|  | LON → SocC → REF | 0.100 | 0.018 | 0.066 – 0.135 |
|  | LON → SubC → REF | 0.098 | 0.016 | 0.067 – 0.129 |
|  | LON → RS → REF | 0.071 | 0.021 | 0.029 – 0.112 |
| LON (T0) → COG (T0) → REF (T1) | LON → ATB | 0.432 | 0.022 | 0.400 – 0.474 |
|  | LON → SocC | 0.294 | 0.020 | 0.255 – 0.334 |
|  | LON → SubC | 0.240 | 0.027 | 0.187 – 0.292 |
|  | LON → RS | 0.577 | 0.024 | 0.529 – 0.625 |
|  | LON → REF | 0.086 | 0.037 | 0.014 – 0.158 |
|  | ATB → REF | 0.210 | 0.029 | 0.154 – 0.267 |
|  | SocC → REF | 0.069 | 0.033 | 0.005 – 0.133 |
|  | SubC → REF | 0.306 | 0.023 | 0.260 – 0.351 |
|  | RS → REF | 0.086 | 0.024 | 0.039 – 0.134 |
|  | LON → ATB → REF | 0.091 | 0.016 | 0.062 – 0.123 |
|  | LON → SocC → REF | 0.041 | 0.015 | 0.023 – 0.079 |
|  | LON → SubC → REF | 0.073 | 0.011 | 0.054 – 0.095 |
|  | LON → RS → REF | 0.050 | 0.015 | 0.020 – 0.080 |
| LON (T0) → COG (T1) → PER (T1) | LON → ATB | 0.536 | 0.021 | 0.494 – 0.577 |
|  | LON → RS | 0.686 | 0.024 | 0.639 – 0.733 |
|  | LON → PER | 0.089 | 0.047 | –0.003 – 0.180 |
|  | ATB → PER | 0.698 | 0.036 | 0.628 – 0.767 |
|  | RS → PER | 0.228 | 0.031 | 0.167 – 0.289 |
|  | LON → ATB → PER | 0.378 | 0.029 | 0.318 – 0.432 |
|  | LON → RS → PER | 0.156 | 0.025 | 0.107 – 0.205 |
| LON (T0) → COG (T0) → PER (T1) | LON → ATB | 0.432 | 0.022 | 0.400 – 0.474 |
|  | LON → RS | 0.577 | 0.024 | 0.529 – 0.625 |
|  | LON → PER | –0.029 | 0.046 | –0.119 – 0.061 |
|  | ATB → PER | 0.472 | 0.034 | 0.406 – 0.539 |
|  | RS → PER | 0.148 | 0.030 | 0.089 – 0.207 |
|  | LON → ATB → PER | 0.204 | 0.021 | 0.164 – 0.246 |
|  | LON → RS → PER | 0.085 | 0.020 | 0.046 – 0.126 |
| REF (T0) → COG (T1) → LON (T1) | REF → ATB | 0.254 | 0.010 | 0.234 – 0.274 |
|  | REF → SocC | 0.213 | 0.009 | 0.195 – 0.231 |
|  | REF → SubC | 0.280 | 0.013 | 0.255 – 0.305 |
|  | REF → LON | 0.051 | 0.009 | 0.033 – 0.069 |
|  | REF → ATB → LON | 0.060 | 0.006 | 0.051 – 0.071 |
|  | REF → SocC → LON | 0.024 | 0.005 | 0.015 – 0.033 |
|  | REF → SubC → LON | 0.014 | 0.004 | 0.005 – 0.022 |
| REF (T0) → COG (T0) → LON (T1) | REF → ATB | 0.186 | 0.011 | 0.164 – 0.208 |
|  | REF → SocC | 0.165 | 0.010 | 0.146 – 0.185 |
|  | REF → SubC | 0.191 | 0.013 | 0.166 – 0.216 |
|  | REF → LON | 0.026 | 0.009 | 0.008 – 0.044 |
|  | REF → ATB → LON | 0.034 | 0.004 | 0.027 – 0.041 |
|  | REF → SocC → LON | 0.013 | 0.003 | 0.007 – 0.019 |
|  | REF → SubC → LON | 0.011 | 0.003 | 0.004 – 0.020 |
| PER (T0) → COG (T1) → LON (T1) | PER → ATB | 0.146 | 0.009 | 0.128 – 0.164 |
|  | PER → LON | –0.001 | 0.008 | –0.015 – 0.015 |
|  | PER → ATB → LON | 0.039 | 0.003 | 0.033 – 0.046 |
| PER (T0) → COG (T0) → LON (T1) | PER → ATB | 0.169 | 0.009 | 0.152 – 0.187 |
|  | PER → LON | 0.003 | 0.007 | –0.012 – 0.017 |
|  | PER → ATB → LON | 0.038 | 0.003 | 0.032 – 0.044 |

Significant effects are those where 95%CI does not include the zero value

Results were adjusted for age, gender, the level of education, employment status, monthly income, a lifetime history of psychiatric treatment, substance use in the preceding month (except for nicotine and alcohol), baseline depressive and anxiety symptoms

*Note:* ATB, attributional biases; COG, cognitive processes; LON, loneliness; PER, ideas of persecution; REF, ideas of reference; RS, rejection sensitivity; SocC, social cognition problems; SubC, subjective cognition problems; T0, baseline measures; T1, follow-up measures

**Figure S1.** The flow diagram of study participants.


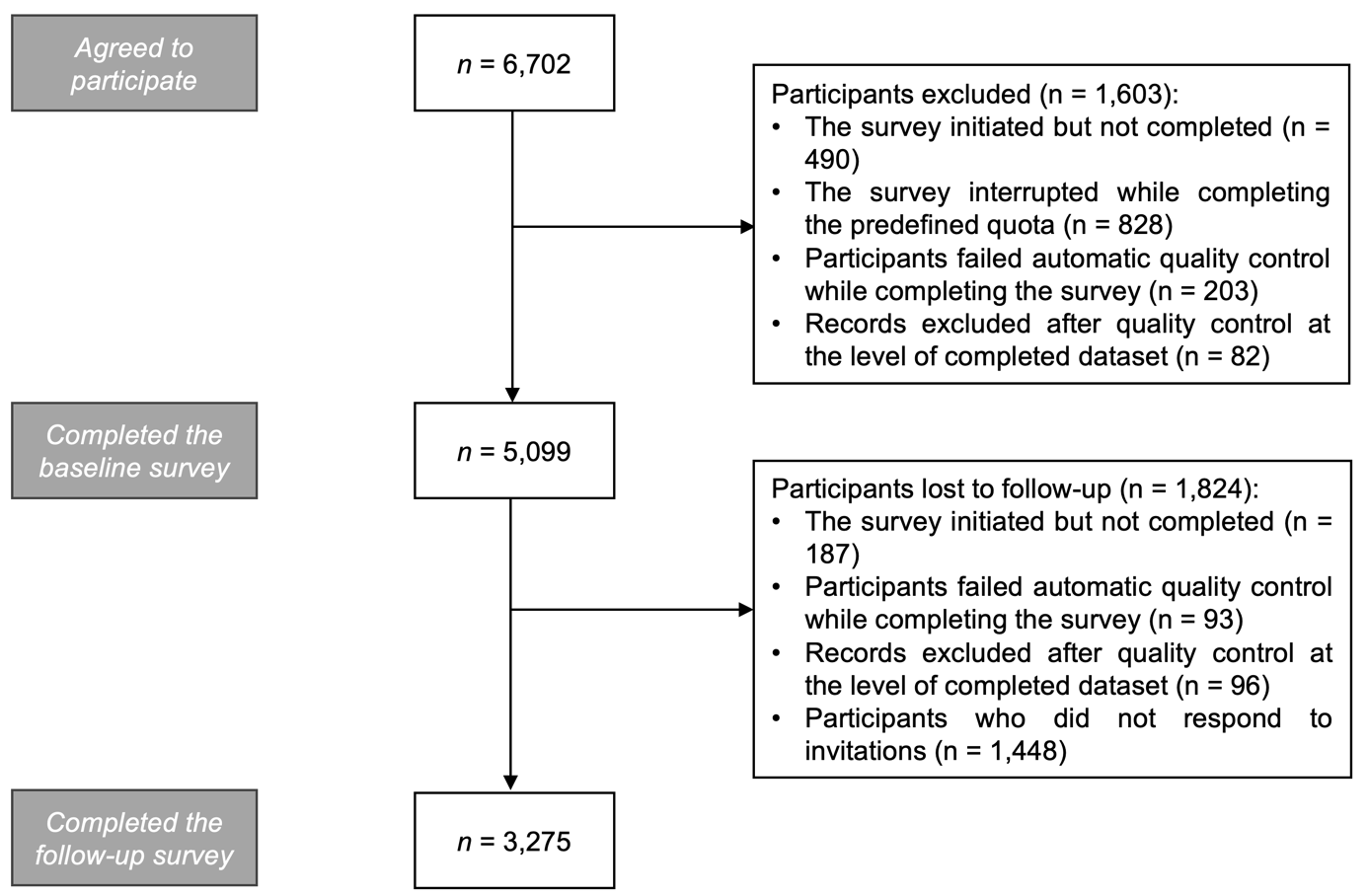


**Figure S2.** Bootstrapped differences between cross-lagged edges. Significant differences between specific edges are marked with black boxes.

**Figure S3.** Bootstrapped differences in out-strength centrality metrics. Significant differences are marked with black boxes.

**Figure S4.** Bootstrapped differences in in-strength centrality metrics. Significant differences are marked with black boxes.

**Figure S5.** Stability of strength centrality metrics while retaining various proportions of cases.

**Figure S6.** Bootstrapped 95%CI around edge weights.
